# Congestion Transition on Random Walks on Graphs

**DOI:** 10.3390/e26080632

**Published:** 2024-07-26

**Authors:** Lorenzo Di Meco, Mirko Degli Esposti, Federico Bellisardi, Armando Bazzani

**Affiliations:** 1 Department of Physics and Astronomy, University of Bologna, 40126 Bologna, Italy; lorenzo.dimeco3@unibo.it (L.D.M.); mirko.degliesposti@unibo.it (M.D.E.); federico.bellisardi2@unibo.it (F.B.); 2 INFN Sezione di Bologna, 40127 Bologna, Italy

**Keywords:** Markov processes, master equation, entropic forces

## Abstract

The formation of congestion on an urban road network is a key issue for the development of sustainable mobility in future smart cities. In this work, we propose a reductionist approach by studying the stationary states of a simple transport model using a random process on a graph, where each node represents a location and the link weights give the transition rates to move from one node to another, representing the mobility demand. Each node has a maximum flow rate and a maximum load capacity, and we assume that the average incoming flow equals the outgoing flow. In the approximation of the single-step process, we are able to analytically characterize the traffic load distribution on the single nodes using a local maximum entropy principle. Our results explain how congested nodes emerge as the total traffic load increases, analogous to a percolation transition where the appearance of a congested node is an independent random event. However, using numerical simulations, we show that in the more realistic case of synchronous dynamics for the nodes, entropic forces introduce correlations among the node states and favor the clustering of empty and congested nodes. Our aim is to highlight the universal properties of congestion formation and, in particular, to understand the role of traffic load fluctuations as a possible precursor of congestion in a transport network.

## 1. Introduction

Modeling city mobility is a crucial aspect of planning future mobility infrastructures and implementing governance policies for sustainable mobility in future smart cities [1,2]. A comprehensive microscopic model to simulate urban mobility must delineate individual mobility demand [3,4], simulate the decision-making mechanisms of individuals to determine mobility strategies, and consider the effects of physical interactions on transport networks [5].

This formidable task requires an enormous amount of data to set up the model parameters and sophisticated methodologies to analyze the simulation results and detect the control parameters of the system. Modeling traffic dynamics has revealed some universal features, such as stop-and-go congestion when vehicle density surpasses a certain threshold [6], which do not depend on the specifics of vehicle interaction. However, the emergence of congestion in an urban transport system is a different phenomenon [7,8], as the dynamics at intersection points become more significant than the dynamics on the roads. Previous studies have proposed percolation theory as a key to understanding the emergence of congestion in a traffic network [9,10,11]. The very definition of congestion on a road network can be approached from different perspectives: individual and macroscopic. The individual perception is mainly influenced by changes in the average velocity of mobility paths, and the uncertainty of travel time can be fundamental in understanding individual behavior in urban mobility [12,13].

Conversely, the macroscopic approach considers the performance of the entire transport system by introducing a macroscopic fundamental diagram [14] and modeling the network dynamics. In this work, we propose a reductionist approach to model transport network dynamics and study congestion formation under stationary conditions by employing a random process on a graph, where each node represents a location, and the link weights (i.e., the transition rates to move from one node to another) are related to the statistical distribution of mobility paths on the network [15] and individual mobility demand. Our aim is to highlight the universal features of congestion transition and the role of traffic load fluctuations.

To this end, we introduce a reductionist model using a random walk on a graph based on two assumptions shared by queue models of transport networks: the existence of a maximum flow rate and a maximum capacity for a road. In the thermodynamic limit, where both the number of nodes and particles tend to infinity but with a fixed ratio, fluctuations remain finite, and an average field approach is not suitable to describe the macroscopic evolution of the system. To highlight the effects of traffic fluctuations, we explicitly study a transport network in a balanced condition where the average incoming and outgoing flows at each node are equal. This assumption reflects the existence of a Wardrop equilibrium [16,17] for urban traffic, where mobility paths distribute to optimize the use of the transport network.

The existence of a maximum flow rate and a finite capacity for each node implies that any displacement involves a maximum number of particles and is possible only if the number of particles in the destination nodes is smaller than the maximum capacity [18,19].

Our main assumption regarding the dynamics is that, in a stationary state, traffic fluctuations can be modeled by a Markov process [20,21]. We recall indeed that a coarse-grained description of a chaotic dynamical system justifies a stochastic approach. The Markov property, indicating short correlations in traffic fluctuations among connected nodes, is likely justified if the mobility demand is distributed across the urban fabric due to city complexity [2].

In the approximation of the single-step process, we can characterize the traffic load distribution of the nodes according to a local maximum entropy principle [22,23]. The application of the entropy concept to understand the statistical laws of urban mobility has been proposed in previous works [24,25,26] using a big-data approach. We extend this result to show how entropy principles allow the study of properties of non-equilibrium states near a stationary state of the transport network model [27,28,29].

We are able to study how the congestion transition can be detected from the fluctuation statistics of the node traffic loads, whose variance reaches a maximum when a peak at the congested nodes appears in the traffic load distribution, but there is no singularity in the thermodynamic limit. Using numerical simulations, we show that macroscopic congestion in the network, due to the emergence of a congested macroscopic cluster, can be accurately explained by a percolation transition, where the appearance of a congested node is an independent event. However, in the more realistic case of synchronous dynamics for the transport network, we demonstrate the appearance of entropic forces [30] that tend to cluster empty and congested nodes, increasing the size of the congested clusters even before the formation of macroscopic congestion. The existence of small congested clusters can introduce significant variance in the travel time distribution for individual paths, and our results suggest that this distribution can be used to characterize congestion formation before the percolation transition.

The paper is organized as follows: In Section 2, we discuss how to use nonlinear stochastic Markov systems as dynamical models of a transport network near a stationary state. In the Section 3, we illustrate the properties of random walks on graphs as models of transport networks and introduce an entropy-based approach to study traffic load fluctuations. In Section 4, we study congestion formation for a simple transport network model. In Section 5, we compare the analytical results with numerical simulations. Finally, we present some conclusions and perspectives.

## 2. Methods: Modeling Transport Network as Markov Processes

Urban traffic is the consequence of individual mobility demand to move from origins to destinations (OD) [3,4]. However, the complex structure of modern cities [31] has ubiquitously distributed activities across the urban fabric, making it extremely difficult to model human mobility using an origin–destination paradigm without collecting information at the individual level [5]. Moreover, the realization of mobility paths in the transport network [7,8] results from both physical interactions (traffic dynamics) and unpredictable individual decisions (free will) in route choice. The statistical physics approach offers a potential solution by using stochastic dynamical models related to a coarse-grained description of traffic dynamics, particularly when the macroscopic and mesoscopic states of traffic do not depend on the details of individual dynamics. The unpredictable features of individual mobility demand justify the use of random dynamical models to simulate mobility on transport networks and the application of the maximum entropy principle (MEP) [22,23] to study the properties of stationary states and the congestion transition. The existence of a *mobility energy* (i.e., travel time budget) is consistent with available data on individual mobility and suggests that mobility in a homogeneous transport network is characterized by an exponential path length distribution [24,25]. These observations suggest that a stochastic model for urban traffic can accurately describe the statistical properties of stationary states but may be inadequate for modeling a system out of equilibrium, where the complexity of urban mobility related to individual behavior could emerge.

The congestion formation on an urban road network depends on two main local features of traffic dynamics: the existence of a finite traffic flow rate at intersections [18] and of the maximal road capacity. The finite flow rate causes the onset of queues at intersections when the incoming flow increases and the maximal road capacity creates gridlock. A microscopic approach that simulates the dynamics of the single mobility paths requires a significant amount of information on the individual mobility demand, or it is based on the synthetic data obtained according to a priori assumptions (e.g., using optimized algorithms to compute the mobility paths from the OD mobility demand). Data on the individual mobility paths are difficult to obtain due to privacy concerns, and the use of synthetic data has the problem of not intruding bias in the system. Being interested in the reconstruction of the traffic flow dynamics along the roads, the necessary information is the measure of the probabilities, $\pi_{ij}$, to observe a path moving between the roads, j→i. The transition probabilities, $\pi_{ij}$, do not require the whole knowledge of the path distribution, but they could be used to directly measure at the intersections. Then, by using a continuity argument one can compute the average traffic flows and highlight the existence of critical situations. However, if traffic distributes on the road network to avoid critical conditions (i.e., we have a balanced condition between average incoming and outgoing flows on each road), then the congestion can be triggered by traffic fluctuations when the traffic load is near to a critical value [32]. To study this effect, one introduces a stochastic process at the intersections that distributes the flows according the transition probabilities, neglecting the correlations among the not directly connected roads. The basic assumption is that each individual could be considered an independent particle and the correlation of traffic fluctuations decays rapidly with distance. This condition appears to be satisfied in modern cities, where mobility has strong random components [33]. If the only knowledge on the traffic dynamics is represented by the transition probabilities, $\pi_{ij}$, and one simulates the particle dynamics according to a random walks on graphs, the Markov condition is consistent with a maximum entropy principle, since this is the stochastic process that maximizes the information entropy of the particle trajectories (i.e., codes by the sequence of nodes) distribution with the constraints that the conditional probabilities, $\pi_{ij}$, for a single evolution step are given [34]. Therefore, random walks on graphs are the models that simulate traffic dynamics, maximizing the information entropy when the available knowledge is the transition probability matrix, $\pi_{ij}$, at road intersections. We also remark that the stationary distribution is completely determined by the transition probabilities so that the random walk models are able to characterize the onset of congestion and the stationary congested road distribution due to traffic fluctuations when traffic load increases. However, the traffic evolution during transient states may depends on long range spatial correlations. The application of a random walk model to study transient states is justified only if the microdynamics is strongly chaotic. Since the daily traffic load has strong periodic components, it is reasonable to assume that each individual tries to optimize their daily mobility cost. Then, one expects that mobility paths distributed on the transport network to create a Wardrop equilibrium with respect to travel time cost. Moreover, under normal conditions, mobility paths can be considered independent of traffic load (i.e., individuals do not change their mobility strategies).

Using a reductionist perspective, we introduce a mathematical description of a transport network using a graph where the nodes i=1,…,M represent either roads or intersections (in our case), and sometimes bus/train stations or generic transport facilities, and the links distribute the traffic load. Each node *i* is characterized by an internal state $n_{i}$ (the traffic load), and the flow $\Phi_{ij}\left(n_{i},n_{j}\right)$ between node *j* and node *i* depends on the node states (Markov field model) [20]. The existence of a maximum flow rate implies that the total flow, $\Phi_{j}$, outgoing from the *j* node satisfies

$$\sum_{i}\Phi_{ij}=\Phi_{j}\leq \Phi_{j}^{\max}.$$

Let Δt be the evolution time scale. If, for a given traffic load, $n_{j}\leq \Phi^{\max}\Delta t$, we have free flow dynamics; conversely, we have queue formation at node *j*, and the crossing time increases proportionally to the queue length (i.e., the traffic load $n_{j}$). Queue formation depends on the total traffic load on the transport network, resulting in a simple fundamental diagram, as the average velocity, $\bar{v}$, decreases with the total traffic load, *N*. When the load, $n_{j}$, of a node approaches the maximum capacity, $n^{\max}$, unavoidable traffic fluctuations can induce gridlock, drastically reducing incoming flows. In this case, the travel time may increase non-linearly with the total traffic load, as it depends on how the congestion spreads in the network [8]. Assuming the existence of a stationary state in a transport network, congestion formation is triggered by traffic fluctuations.

If there are no long-range spatial correlations in the mobility path distribution, the traffic dynamics at a stationary state can be approximated by a Markov random process with transition probabilities $\pi_{ij}$ (i.e., a non-linear random walk on a graph). The OD nature of urban mobility is not relevant for defining the stationary state when the correlation between the paths of two ’particles’ in the same node is negligible (i.e., the probability that two randomly chosen particles in the same node have similar destinations is very small). Moreover, one can justify the relaxation process to a stationary state and study the effect of fluctuations by approximating the dynamics with a Markov process [21,27]. The average dynamics of the transport network reads

(1) $${\dot{n}}_{i}=\sum_{j}\left[\Phi_{ij}\left(n_{i},n_{j}\right)-\Phi_{ji}\left(n_{j},n_{i}\right)\right]+s_{i}\left(t\right),$$

where $s_{i}\left(t\right)$ represent the particle sources or the sinks present in the system that modulate the total traffic load. To study the stationary states we set $s_{i}=0$ and the traffic load $\sum_{i}n_{i}=N$ is constant. An average equilibrium solution satisfies

(2) $$\sum_{j}\left[\Phi_{ij}\left(n_{i}^{*},n_{j}^{*}\right)-\Phi_{ji}\left(n_{j}^{*},n_{i}^{*}\right)\right]=0\;\sum_{j}n_{j}^{*}=N,$$

and the congestion transition occurs when the stationary solution becomes unstable and a new solution emerges with $n_{j}=n^{\max}$ for a subset of nodes (the congested nodes). When the congested nodes form a giant cluster in the transport network, we say that the whole network is in a congested state.

### Characterizing the Stationary State

The equilibrium states are determined by the stationary flows among the connected nodes. By definition, $\pi_{ij}$ is the probability that a particle performs the transition j→i and does not depend on the traffic load fluctuations. $\pi_{ij}$ is a stochastic matrix, and in a free traffic condition one can set $\Phi_{ij}\propto \pi_{ij}n_{j}$. However, a maximum flow rate, $\phi_{i}^{\max}$, and a maximum node capacity, $n_{i}^{\max}$, exist (i.e., $\Phi_{ij}=0$ if $n_{i}\geq n_{i}^{\max}$). A possible definition for the flows $\Phi_{ij}\left(n_{i},n_{j}\right)$ is

(3) $$\Phi_{ij}\left(n_{i},n_{j}\right)=\pi_{ij}\phi_{j}^{\max}c\left(n_{i}/n_{i}^{\max}\right)\phi \left(n_{j}\right),$$

where the function $\phi \left(n_{j}\right)\in \left[0,1\right]$ is assumed to be monotonic, increasing with an initial linear dependence and an asymptotic limit, $\lim_{n\to \infty }\phi \left(n\right)=1$ (i.e., we do not consider a reduction of the outgoing flow when the node is congested, assuming that the dynamics at intersections is weakly affected by the road congestion), and the capacity function $c\left(n_{i}/n_{i}^{\max}\right)\in \left[0,1\right]$ is a threshold function that drops down to zero when $n_{i}\geq n_{i}^{\max}$. The flow and the capacity functions simulate the effect of particle interactions that affect the traffic dynamics. In the case of urban road network, the flow function, ϕ(n), is simulated by the traffic dynamics at intersections. As a consequence of definition (3), the transport network has a flow-density fundamental diagram when we increase the average traffic load for each node (cfr. Section 5). The equilibrium solution (2) follows from the condition

(4) $$\sum_{j}\pi_{ij}\phi_{j}^{\max}c\left(n_{i}/n_{i}^{\max}\right)\phi \left(n_{j}\right)=\sum_{j}\pi_{ji}\phi_{i}^{\max}c\left(n_{j}/n_{j}^{\max}\right)\phi \left(n_{i}\right).$$

If there exists a solution with $n_{i}\leq n_{i}^{\max}$, the traffic load is sustainable, otherwise we have congested states with some nodes at maximum capacity. In the case of a low traffic load, we expect $\phi \left(n_{j}\right)=\alpha n_{j}$ and $c(n_{i}/n_{i}^{\max})=1$ and Equation (4) simplifies

$$\sum_{j}\pi_{ij}\phi_{j}^{\max}n_{j}^{*}=\phi_{i}^{\max}n_{i}^{*}.$$

$n_{i}^{*}$ turns out to be the stationary eigenvector of the stochastic matrix $\pi_{ij}$, and the equilibrium state is stable since all the other eigenvalues, λ, of the Laplacian matrix of the network,

(5) $$L_{ij}=\delta_{ij}\phi_{i}^{\max}-\pi_{ij}\phi_{j}^{\max},$$

have a negative real part if the network is connected [35]. When the traffic load increases, the flow function ϕ(n)→1, and we have to consider a self-consistent approach assuming $c(n_{i}/n_{i}^{\max})=1$ (i.e., no congestion in the network). We compute the null eigenvector $\phi_{i}^{*}$,

$$\sum_{j}\pi_{ij}\phi_{j}^{\max}\phi_{j}^{*}-\phi_{i}^{\max}\phi_{i}^{*}=0,$$

and we look for the solutions $n_{i}^{*}$ of the system,

$$\phi \left(n_{i}^{*}\right)\propto \phi_{i}^{*}\;\sum_{i}n_{i}^{*}=N.$$

We study the stability of the equilibrium solution in the presence of perturbations, $\delta n_{j}$, such that $\sum_{j}\delta n_{j}=0$ (i.e., the total traffic load is constant). The stability character depends on the derivative $\phi^{\prime }=d\phi /dn$: if $\phi^{\prime }\left(n_{j}^{*}\right)>0$∀j, then the linearized system,

$$\delta {\dot{n}}_{i}=-\sum_{j}L_{ij}\phi^{\prime }\left(n_{j}^{*}\right)\delta n_{j},$$

is still associated with a Laplacian matrix, and the eigenvalues all have a negative real part on the invariant subspace, $\sum_{j}\delta n_{j}=0$. When $\phi^{\prime }\left(n_{j}^{*}\right)\to 0$, the system tends to a neutral stability. However, if the equilibrium traffic load, $n_{i}^{*}$, increases up tp $n_{i}^{\max}$, congested states appear due to the the congested function $c(n_{i}/n_{i}^{\max})$ in definition (3). Assuming $n_{i}/n_{i}^{\max}≃1$, we can simplify Equation (4), letting $\phi \left(n_{j}^{*}\right)≃\phi_{j}^{max}$ (i.e., before the congestion the roads express their maximum flow rate), the equilibrium condition reads

$$\sum_{j}\pi_{ij}\phi_{j}^{max}c\left(n_{i}/n_{i}^{\max}\right)=\sum_{j}\pi_{ji}\phi_{i}^{\max}c\left(n_{j}/n_{j}^{\max}\right).$$

Therefore, the solution has the form $c\left(n_{i}^{*}/n_{i}^{max}\right)\propto c_{i}$, where $c_{i}$ is the null eigenvector of the Laplacian matrix,

(6) $$L_{ij}^{\prime }=\sum_{k}\pi_{ik}\phi_{k}^{max}\delta_{ij}-\pi_{ji}\phi_{i}^{\max},$$

(cfr. Equation (5)) with the constraints

$$\sum_{i}n_{i}^{*}=N\;\mathrm{and}\;n_{i}^{*}<n_{i}^{\max}.$$

By increasing the traffic load *N*, $n_{i}^{*}\to n_{i}^{\max}$ for the nodes corresponding to the smallest values of $c_{i}$, and the congestion will start from these nodes. To study the stability of the solution, we consider the linearized system, i.e.,

$$\delta {\dot{n}}_{i}=\sum_{j}L_{ij}^{\prime }c^{\prime }\left(n_{j}^{*}\right)\delta n_{j}\;c^{\prime }\left(n_{j}^{*}\right)=\frac{dc}{dn_{j}}\left(n_{j}^{*}\right),$$

and when $c^{\prime }\left(n_{j}^{*}\right)<0$, we have a stable congested state. We observe that if $\Phi_{j}^{\max}$ is the stationary eigenvector of the stochastic matrix $\pi_{ij}$, we have $L_{ij}=L_{ij}^{\prime }$, and the vectors $\phi_{i}^{*}$ and $c_{i}$ are constant (i.e., at the equilibrium states, $\Phi_{i}/\phi_{i}^{\max}$ and $n_{i}/n_{i}^{\max}$ have the same value for all the nodes).

A possible interpretation is that individuals use the transport network in such a way that the traffic load is distributed, making the nodes equivalent from a dynamical point of view. This condition could reflect the emergence of a Wardrop equilibrium, where the paths distribution evolves to define a transition matrix, $\pi_{ij}$, which makes all nodes equivalent in terms of congestion formation. In other words, any change in the path distribution would make a node more susceptible to congestion.

Since the nodes are equivalent, congestion formation depends on the presence of traffic fluctuations that prevent the transport system from reaching its maximum flow rate, making congestion a dynamical stationary state. When a node *i* becomes congested ($n_{i}\geq n_{i}^{\max}$), its incoming flow is null, rendering some links, $\pi_{ij}$, ineffective. Consequently, the connected nodes, *j*, have a higher probability of becoming congested, whereas node *i* can exit the congested state since its flow $\Phi_{i}>0$. Thus, we do not have an equilibrium state with fixed congested nodes, but rather a stationary dynamical state where congestion moves across the network in regions of almost congested nodes.

The rise of congestion due to traffic load fluctuations is not described by the average dynamics (1), and the fluctuations define the traffic load’s stationary distribution on the transport network.

## 3. Random Walk on Graphs as Models of Transport Networks

To study the physics of traffic fluctuations, we propose simple models based on random walks on graphs [35]. According to the previous section, we associate a graph with a transport network where the transition probabilities, $\pi_{ij}$, define the link weights. This allows us to define a Markov process in which the ’particles’ move randomly according to the transition probabilities. In this way, it is possible to study the fluctuation statistics at stationary states and congestion formation.

To perform an analytical approach, we simplify model (3) by defining the flow function ϕ(n)=Θ(n), where Θ(n) is the Heaviside function (i.e., Θ(n)=1 if n≥1 and Θ(0)=0) and the capacity function $c\left(n\right)=\Theta \left(n^{\max}-n\right)$, where all the nodes have the same maximum capacity, $n_{max}$. We refer to the vector $\vec{n}$, whose components $n_{i}\geq 0$i=1,...,M give the number of particles at node *i* as the dynamical state of the network, and we define $|\vec{n}|=\sum_{i}n_{i}=N$ as the traffic load of the transport network. In the one-step process approximation, the evolution of the distribution function $\rho (\vec{n},t)$ is given by the master equation

(7) $$\frac{1}{M}\dot{\rho }\left(\vec{n},t\right)=\sum_{\left(i,j\right)}\left[E_{i}^{-}E_{j}^{+}\Phi_{ij}\left(n_{i},n_{j}\right)-\Phi_{ji}\left(n_{j},n_{i}\right)\right]\rho \left(\vec{n},t\right),$$

where we set the transition rates,

(8) $$\Phi_{ij}\left(n_{i},n_{j}\right)=\Theta \left(n_{j}\right)\Theta \left(n^{\max}-n_{i}\right)\pi_{ij},$$

and the sum runs over all the possible ordered couples (i,j).

We remark that the one-step process implies an instantaneous update of information after each movement. This is a non-physical assumption for a transport network, and we also consider synchronous dynamics where all nodes evolve simultaneously, and the information of the network state is updated after the movement of all nodes. In this case, the master equation becomes more complicated and an analytical approach is not feasible. In Appendix A, we show how to write the incoming and outgoing flows for a generic node (see Equation (A5)).

We observe that if j≠i, we have the identity

$$E_{i}^{-}E_{j}^{+}\pi_{ij}\Theta \left(n_{j}\right)\Theta \left(n^{\max}-n_{i}\right)\rho \left(\vec{n}\right)=\Theta \left(n_{i}\right)\Theta \left(n^{\max}-n_{j}\right)\pi_{ij}\rho \left(\vec{n}+{\hat{e}}_{j}-{\hat{e}}_{i}\right),$$

where we set $\rho (\vec{n})=0$ for any non-physical state and we write the master equation in the form

(9) $$\frac{1}{M}\dot{\rho }\left(\vec{n},t\right)=\sum_{\left(i,j\right)}\Theta \left(n_{i}\right)\Theta \left(n^{\max}-n_{j}\right)\left[\pi_{ij}\rho \left(\vec{n}+{\hat{e}}_{j}-{\hat{e}}_{i},t\right)-\pi_{ji}\rho \left(\vec{n},t\right)\right].$$

### 3.1. Equilibrium State in the Case of Detailed Balance

We are interested in the stationary distribution, $\rho_{s}\left(\vec{n}\right)$, of the master Equation (9). Let $\vec{p}$ be the null right eigenvector of the Laplacian matrix of the weighted network (i.e., $p_{i}$ is the probability to observe a particle in node *i* according to the transition rates (8)),

(10) $$\sum_{j}\pi_{ji}p_{i}-\pi_{ij}p_{j}=0\;\sum_{j}p_{j}=1\;p_{j}>0.$$

We look for a stationary distribution of the form

(11) $$\rho_{s}\left(\vec{n}\;\right)=\left\{\begin{array}{cc} {\left[C_{N}^{\;n^{\max}}\right]}^{-1}\left(\vec{p}\;\right)\prod_{k=1}^{M}p_{k}^{n_{k}} & \;|\vec{n}|=N\;n^{\max}\geq n_{i}\geq 0\\ 0 & \mathrm{otherwise}. \end{array}\right.$$

where $C_{N}^{n^{*}}\left(\vec{p}\;\right)$ is the normalizing constant (i.e., the partition function), and it is related to the Helmholtz Free Energy,

(12) $$F\left(\vec{p}\;\right)=-\ln C_{N}^{\;n^{\max}}\left(\vec{p}\;\right)=-\ln\sum_{|\vec{n}|=N}^{n^{\max}}\prod_{i=1}^{M}p_{i}^{n_{i}},$$

where the sum runs over the physical states $n_{i}\leq n^{\max}$ (see Equation (A1) in Appendix A for some analytical estimates in the case of $n^{\max}\gg 1$). The stationary condition reads

(13) $$\sum_{\left(i,j\right)}\Theta \left(n_{i}\right)\Theta \left(n^{*}-n_{j}\right)\left[\pi_{ij}\frac{p_{j}}{p_{i}}-\pi_{ji}\right]\rho_{s}\left(\vec{n}\right)=0,$$

which has to be satisfied for all the physical states, $\vec{n}$. The main difficulty of obtaining an analytical solution of Equation (13) is the presence of empty and congested nodes at the same time. However, if the micro-dynamics satisfies a detailed balance (DB) condition,

(14) $$\pi_{ij}p_{j}=\pi_{ji}p_{i},$$

the distribution (11) is the stationary solution of the master Equation (9). The DB condition means that, in the stationary state, the probability to observe a path between the nodes j→i is the same if we consider the reverse displacement i→j. We remark that the stationary distribution, $\vec{p}$, depends on the transition matrix, $\pi_{ij}$, but different transition matrices have the same distribution. The DB condition associates uniquely the transition matrix to the distribution $\vec{p}$. If we introduce the Gibbs Entropy,

(15) $$S\left[\rho \left(\vec{n}\;\right)\right]=-\sum_{|\vec{n}|=N}^{n^{\max}}\rho \left(\vec{n}\;\right)\ln\rho \left(\vec{n}\;\right),$$

the DB condition allows one to apply a maximum entropy principle (MPE) to compute the stationary distribution. Let ${\bar{n}}_{i}$ be the mean load per node,

(16) $${\bar{n}}_{i}=\sum_{|\vec{n}|=N}^{n^{\max}}n_{i}\rho_{s}\left(\vec{n}\;\right)\;i=1,...,M.$$

The Gibbs entropy (15) is maximum for the distribution (11) when the probabilities, $p_{i}$, satisfy the constraints (16). The thermodynamic approach allows one to characterize the statistical properties of the stationary state without considering the dynamics (7). An explicit computation of the entropy for a distribution of the form (11) gives

$$S\left(\vec{p}\;,N\right)=\ln C_{N}^{\;n^{\max}}\left(\vec{p}\;\right)-\sum_{|\vec{n}|=N}^{n^{\max}}\rho_{s}\left(\vec{n}\;\right)\sum_{i}n_{i}\ln p_{i}=-F\left(\vec{p}\;\right)-\sum_{i}{\bar{n}}_{i}\ln p_{i},$$

and we get

(17) $$\frac{\partial S}{\partial p_{i}}=-\frac{\partial }{\partial p_{i}}F\left(\vec{p}\;\right)-\frac{{\bar{n}}_{i}}{p_{i}}.$$

The extremality condition reads

$${\bar{n}}_{i}=-p_{i}\frac{\partial }{\partial p_{i}}F\left(\vec{p}\;\right),$$

and it is verified by the distribution (11). When one increases the total traffic load, *N*, we observe that the distribution (11) is peaked on the marginal states where the nodes with the greater $p_{i}$ are congested, $n_{i}=n^{\max}$, and the other nodes are empty. This means that the nodes with greater $p_{i}$ are hot spots for the congestion spreads and the mitigation policies have to reduce the traffic load on these nodes by redistributing the mobility paths. The situation is different when one considers a homogeneous transport network where $p_{i}=M^{-1}$ for all the nodes. Among all the possible distributions of the form (11), this provides the maximum value for the Gibbs entropy and the congestion formation is triggered by the traffic fluctuations since ${\bar{n}}_{i}=N/M$ for any node.

To study the relation between entropy and traffic load fluctuations, we compute the Hessian matrix of the entropy with respect to the variation of $p_{i}$,

(18) $$\begin{array}{cc} \; & p_{j}p_{i}\frac{\partial^{2}S}{\partial p_{j}\partial p_{i}}=p_{j}p_{i}\frac{\partial }{\partial p_{j}}p_{i}^{-1}\left(-p_{i}\frac{\partial }{\partial p_{i}}F\left(\vec{p}\right)-{\bar{n}}_{i}\right)=\\ \; & =-p_{j}\frac{\partial }{\partial p_{j}}{\left[C_{N}^{\;n^{*}}\right]}^{-1}\sum_{|\vec{n}|=N}^{n^{\max}}n_{i}\prod_{k=1}^{M}p_{k}^{n_{k}}=-\left(\langle n_{i}n_{j}\rangle -{\bar{n}}_{j}{\bar{n}}_{i}\right), \end{array}$$

where we have used Equation (17). When the system is in a stationary state, the covariance matrix of the traffic load, $n_{i}$, is the sensitivity of the entropy to the changes of the probability distribution, $p_{i}$. Since the distribution, $p_{i}$, depends on the transition probabilities, $\pi_{ij}$, the previous equation also gives a measure of the sensitivity to the perturbations of the transition matrix. Using the entropy as a measure of the disorder in the system, the result (18) means that when the fluctuations of the traffic load, $n_{i}\left(t\right)$, are large, the system may pass through ordered and disordered states during the evolution. We also have the reciprocity relations that holds in the DB condition,

$$p_{j}\frac{\partial {\bar{n}}_{i}}{\partial p_{j}}=p_{i}\frac{\partial {\bar{n}}_{j}}{\partial p_{i}}=-p_{i}p_{j}\frac{\partial F}{\partial p_{i}\partial p_{j}}\;i\neq j,$$

to define how the average load of different nodes is affected by the change of the stationary probabilities; these relations correspond to the Onsager reciprocal relations.

In the case of a homogeneous network, the fluctuation variance at each node is a key indicator for understanding the rise of congestion. One expects that the fluctuations reduce when the traffic load is low ($n_{i}\ll n^{\max}$) or highly congested ($n_{i}≃n^{\max}$), so that one get a critical value for the total traffic load when the average fluctuation variance is maximum. Using the covariance matrix with $Np_{i}=n_{i}$,

$$\sigma_{ij}\left(N\right)=-n_{i}n_{j}\frac{\partial^{2}S}{\partial n_{j}\partial n_{i}}\left(N\right),$$

we introduce the following definition,

$$\bar{\sigma }\left(N\right)=\frac{1}{M}\mathrm{Tr}\;\sigma =\frac{1}{M}\sum_{i}n_{i}^{2}\frac{\partial^{2}S}{\partial n_{i}^{2}}\left(N\right),$$

and the critical load $N_{c}$ satisfies

(19) $$\frac{\partial \bar{\sigma }}{\partial N}\left(N_{c}\right)=0.$$

Using numerical simulations on a simple traffic network model, we have computed the traffic load variance for the traffic load of single nodes as a function of the average traffic load (see Section 5). The simulations show that the maximum value of the standard deviation is achieved at the critical value of the flow-density fundamental diagram. The critical load, $N_{c}$, corresponds both to the maximum flow in the transport network and to the maximum uncertainty in traffic load distribution. When $N\geq N_{c}$, the congested nodes start to merge in clusters until a macroscopic large cluster emerges in the network. The congestion degree can be related to the dimension of congested clusters, which is the fingerprint of a percolation phase transition for the congestion formation [11].

### 3.2. Non-Equilibrium Stationary States

When the detailed balance condition (14) does not hold, the Markov process to model the vehicle dynamics realizes a non-reversible random walk, i.e., the statistics of the reverse paths on the transport network is not equivalent to the statistics of the original paths in the stationary states. As a consequence, we have the presence of probability stationary currents on the links j→i, defined by

(20) $$J_{ij}\left(\vec{n}\right)=\Theta \left(n_{i}\right)\Theta \left(n^{\max}-n_{j}\right)\left[\pi_{ij}\rho_{s}\left(\vec{n}+{\hat{e}}_{j}-{\hat{e}}_{i},t\right)-\pi_{ji}\rho_{s}\left(\vec{n},t\right)\right],$$

that correspond to net traffic flows moving on the loops of the transport network. In such a case, the MEP cannot be applied [28], but it is possible to maximize a local entropy or apply an entropy production principle [29]. If we consider the ensemble of states $\vec{n}$ with $0\leq n_{j}<n^{\max}$ so that $\Theta (n^{\max}-n_{j})=1$, then the stationary solution satisfies

$$\sum_{j}\left[\pi_{ij}\rho_{s}\left(\vec{n}+{\hat{e}}_{j}-{\hat{e}}_{i},t\right)-\pi_{ji}\rho_{s}\left(\vec{n},t\right)\right]=0,$$

and we recover a solution of the form (11) that maximizes the entropy if restricted to this ensemble (cfr. Equation (13)). Conversely, if one considers the ensemble of states $\vec{n}$ with $0<n_{i}\leq n^{\max}$ and $\Theta (n_{i})=1$ and we get the condition

$$\sum_{i}\left[\pi_{ij}\rho_{s}\left(\vec{n}+{\hat{e}}_{j}-{\hat{e}}_{i},t\right)-\pi_{ji}\rho_{s}\left(\vec{n},t\right)\right]=0,$$

then we have a solution of the form

(21) $$\rho_{s}\left(\vec{n}\;\right)\propto \prod_{k=1}^{M}q_{k}^{-n_{k}},$$

where $\vec{q}$ is the stationary eigenvector of the Laplacian matrix associated with the reverse transition matrix, $\pi_{ij}^{T}$. The solution (21) approximates the stationary distribution in the case of high traffic loads, and it is a maximum entropy solution for the congested states. The distribution (21) is the distribution for the gap dynamics on a congested transport network. In the case of DB, $q_{i}=p_{i}^{-1}$ and the two distributions coincide. Changing the traffic load, the stationary distribution, $\rho_{s}\left(\vec{n}\right)$, changes its form, interpolating the two limit distributions. The congestion transition depends on the probability of intermediate states, where one has both empty and congested nodes.

## 4. Results: Congestion Formation in Balanced Transport Networks

To understand the role of traffic fluctuations in the congestion transition, we consider a homogeneous transport network where all the roads are equivalent, $p_{i}=const.$ Using the simple model (8), this condition corresponds to the balance condition

(22) $$\sum_{i}\pi_{ij}=\sum_{j}\pi_{ij}.$$

For each node, the expected incoming flow is balanced by the expected outgoing flow. This condition is consistent with the existence of Wardrop’s equilibrium in transport systems. We also note that condition (22) does not imply detailed balance, which requires the symmetry of the transition rates, $\pi_{ij}$. The stationary solution can be approximated by a uniform distribution (cf. Equation (11)) for the majority of the microstates, but not for the network states $\vec{n}$, where empty and congested connected nodes are simultaneously present. Due to traffic fluctuations, these states are more probable when the average traffic load is $N/M=n^{\max}/2$, where the probability of empty and congested nodes is equal, so they can play a significant role in the congestion transition.

To study how the node states are distributed in the network, we consider the single-node distribution p(n), defined as the probability to observe a node in the state *n* in the stationary state. In an homogeneous transport network, all the nodes are equivalent so that the marginal distribution for a single node of the stationary distribution, $\rho_{s}\left(\vec{n}\right)$, approximates the traffic load distribution on the network in the thermodynamic limit. The distribution p(n) can be related to the congestion degree by computing the probability of the almost congested states,

$$C\left(\tau ,N\right)≃\sum_{n\geq n^{\max}-n\left(\tau \right)}p\left(n\right),$$

where a suitable n(τ) is a decreasing function of τ.

In the case of low traffic load, $N/M\ll n^{\max}$, it is possible to prove that in the thermodynamics limit the single-node distribution, p(n), is approximated by an exponential distribution,

(23) $$p\left(n\right)≃\frac{1}{\bar{n}}\exp\left(-\frac{n}{\bar{n}}\right)\;\bar{n}=\frac{N}{M},$$

and the congestion probability is exponentially small. Analogously, in the case of high traffic load (i.e., $N≃n^{\max}M$), it is approximated by

(24) $$p\left(n\right)\propto \exp\left(-\frac{n^{\max}-n}{n^{\max}-\bar{n}}\right)\;n\leq n^{\max}$$

(see Appendix A for the proof), which explains why the number of congested nodes, C(τ,N), increases with the traffic load, *N*. The true distribution interpolates the two approximations as the traffic load varies, but this is a continuous process and the distribution does not cross any singularity (see Section 5).

In the case of synchronous dynamics, each node moves one particle but can receive a variable number of particles depending on its connectivity degree. An analytic expression for the stationary distribution $\rho_{s}\left(\vec{n}\right)$ is not available, and we note that synchronous dynamics introduces correlations among the states of connected nodes. Indeed, a node connected to empty nodes has a reduced incoming flow on average, causing its population to decrease. Consequently, network states where empty nodes cluster are favored by synchronized dynamics. Similarly, if a node is connected to a congested node, its outgoing flows are reduced on average, causing its population to increase, and thus congested nodes also tend to cluster. This phenomenon can be interpreted by introducing entropic forces that attract empty and congested nodes in synchronous dynamics. In the last part of Appendix A, we discuss the relationship between synchronous dynamics and the appearance of entropic forces. In Section 5, we study the effect of entropic forces using numerical simulations.

### Single-Node Dynamics

We now consider the following problem:

Is it possible to introduce an effective model for the node dynamics to quantify the effect of entropic forces?

The node state evolution can be modeled by an effective master equation that depends on the neighbor node states. In the one-step process model, it is possible to look for a self-consistent master equation for the distribution p(n). One can consider the balance between the incoming and outgoing flows of a representative node *i* given its state $n\leq n^{\max}$, averaged over all the states of the network. The incoming flow is the transition probability from the state n−1→n so that n≥1 and we get

$$\langle \sum_{j}\pi_{ij}\Theta \left(n_{j}\right)-\sum_{j}\pi_{ji}\Theta \left(n^{\max}-n_{j}\right)\rangle =0.$$

One applies the following estimates

$$\langle \Theta \left(n_{j}\right)\rangle =1-\pi \left(0|n-1\right)p\left(n-1\right)\;\langle \Theta \left(n^{\max}-n_{j}\right)\rangle =1-\pi \left(n^{\max}|n\right)p\left(n\right),$$

where π(0|n−1) is the conditional probability that a neighbor is empty when the node state is $n_{i}=n-1$ and $\pi (n^{\max}|n)$ is the conditional probability that a neighbor is congested when the node state $n_{i}=n$. Using condition (22), we have the equilibrium

(25) $$\left(1-\pi \left(0|n-1\right)\right)p\left(n-1\right)-\left(1-\pi \left(n^{\max}|n\right)\right)p\left(n\right)=0\;0<n\leq n^{\max}.$$

The conditional probabilities may depend on the degree of the node, i.e., the average is the result of an averaging on all the nodes. Equation (25) can be solved recursively using p(0) to normalize the distribution. We remark that the conditional probabilities are estimated from the global dynamical properties of the transport network and measure the correlation between the states of connected nodes. In the one-step process case, the distribution is derived from an MEP, and we expect that the node states are independent so that in a thermodynamic limit π(0|n)=p(0) and $\pi \left(n^{\max}|n\right)=p\left(n^{\max}\right)$. In synchronous dynamics, the stationary distribution does not maximize the entropy, and we expect that π(0|n) is a decreasing function on *n* since the empty nodes tend to cluster and $\pi (n^{\max}|n)$ is an increasing function. These effects are a consequence of the stochastic dynamics when the network state contains empty and congested nodes at the same time and it creates entropic forces that explains the correlation among the connected nodes.

## 5. Results: Numerical Simulations of the Transport Network Model

We have checked the applicability of the analytical estimate by simulating the simplified model (8) using a random network of 500 nodes with average degree d=3 (but the minimum degree is d=2). For a given transition matrix, $\pi_{ij}$, we have computed the stationary solution, $p_{i}$, and we have define the stationary flows $\Phi_{ij}=\pi_{ij}p_{j}$, assuming $\phi^{\max}=1$; we have the balance condition

$$\sum_{j}\Phi_{ij}=\sum_{j}\pi_{ij}p_{j}=p_{i}=\sum_{j}\pi_{ji}p_{i}=\sum_{i}\Phi_{ij}.$$

As previously discussed, this condition means that the flows distribute on the transport network so that the average incoming flows equal the average outgoing flows for each node, simulating an optimal use of the network. The maximum average flow for the whole network is defined by

(26) $${\bar{\Phi }}^{\max}=\frac{1}{M}\sum_{ij}\Phi_{ij},$$

and the maximum node capacity is fixed at $n^{\max}=10$. In the sequel, we refer to this model as the *transport network model*. The balance condition (22) for the flows highlights the role of fluctuations in the emergence of the congested states, but we remark that the transition matrix, $\pi_{ij}$, does not satisfy the DB condition (i.e., $\Phi_{ij}$ is not symmetric). This model is used in all the simulations presented in the sequel.

**Figure 1:**
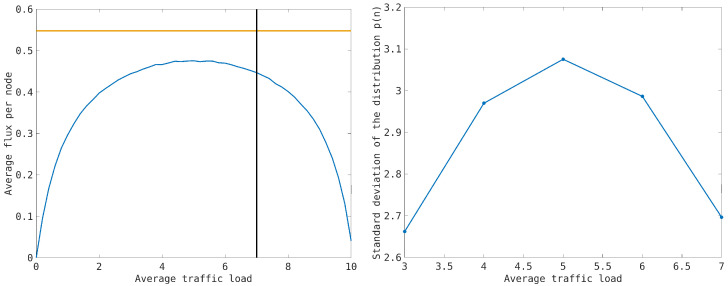
**Left picture**: Fundamental diagram for the transport network model using synchronous dynamics, where the average flow is plotted as a function of the average traffic load. The horizontal line denotes the maximum possible average flow in the network, which is greater than the numerical value achieved at $\bar{n}=n^{\max}/2=5$ due to the presence of empty and congested nodes at any traffic load caused by traffic fluctuations. The vertical line at $\bar{n}=7$ indicates the critical traffic load at which small congested clusters start to merge (see Figure 8). **Right picture**: Standard deviation of the single-node traffic load distribution using the transport model in synchronous dynamics as a function of the average traffic load $3\leq \bar{n}\leq 7$. The maximum value of the standard deviation is obtained at the critical value $\bar{n}=5$ of the traffic load.

In Figure 1, we plot the average flow nodes (26) with respect to the average traffic load per node $\bar{n}$ to get the fundamental diagram for the network [14] that points out that the maximum flow is reached at $\bar{n}=n^{\max}/2$, which is lower than the maximum theoretical value since the presence of the congested nodes and empty nodes due to the fluctuations for any traffic load prevents the network transport capacity from reaching its maximum value. The fundamental diagram shows that the average flow is almost constant for a large fraction of traffic load $3\leq \bar{n}\leq 7$, and it quickly reduces to zero at the limit values $\bar{n}=0$ and $\bar{n}=10$. We observe that the traffic load $\bar{n}=7$, at which the flow rate begins to drop down, coincides with the critical value at which the clusters of congested nodes start to merge (see Figure 8). This value can be considered a precursor of a macroscopic congestion formation.

**Figure 2:**
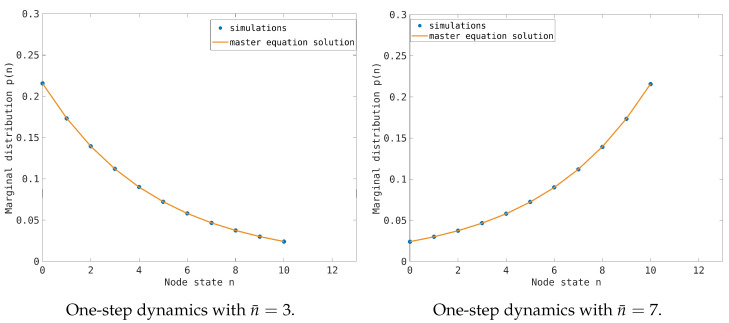

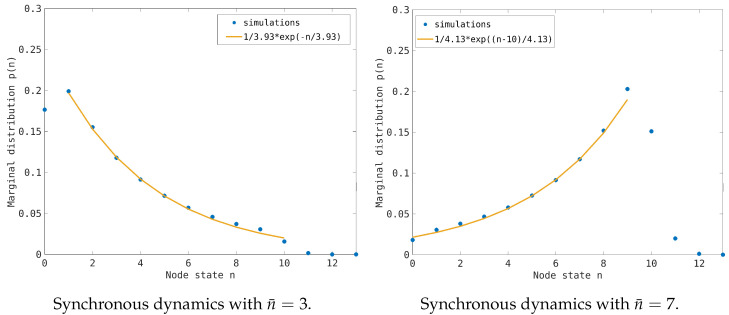
**(Top pictures)**: The dots represent the empirical distributions, p(n), of the node traffic load for the transport network model in the one-step process approximation. The distributions are computed by averaging over $10^{5}$ evolution steps of the model. The maximum node capacity is $n^{\max}=10$, and we consider two average traffic loads: a low traffic load, $\bar{n}=3$ (**left picture**), and a high traffic load, $\bar{n}=7$ (**right picture**). The continuous lines refer to the stationary solution of the single-node dynamics (25). (**Bottom pictures**): The dots represent the empirical distributions of the node traffic load for the transport network model using the synchronous dynamics. The parameters used in the simulations are the same as in the top pictures. The continuous line is an exponential interpolation according to Equations (23) and (24).

To explain the fundamental diagram, we compute the traffic load distribution, p(n), on the network (see Section 4 for the definition) as a function of the average traffic load. The simulation results are shown in Figure 2, where we consider both the one-step process (top pictures) and the synchronous dynamics (bottom pictures). To average out the finite size fluctuations, the empirical stationary distributions are computed by applying an ergodic principle and averaging over $10^{5}$ evolution steps. For the one-step process, we compare the simulation results with the equilibrium distribution for the single-node dynamics (25) and find perfect agreement between the two approaches. The empirical distributions for $\bar{n}=3,7$ are also well approximated by the exponential distributions (23) and (24), as expected by an entropy principle. This is also true in the more realistic case of synchronous dynamics, where we compare the stationary empirical distribution computed by averaging over $10^{5}$ iterations with the exponential approximation for $\bar{n}=3,7$.

The distribution p(n) indicates that, for any traffic load, the probability of observing both empty and congested nodes reduces the traffic flow on the network. Over the critical threshold $\bar{n}=5$, the distribution is peaked at the congested nodes. However, the emergence of congestion in the network is a continuous process, and no singular behavior is observed in the distribution.

In the synchronous evolution, we observe that, for a low traffic load, the empty state is underrepresented compared to the exponential interpolation, and states with traffic load $n>n^{\max}$ are present.

**Figure 3:**
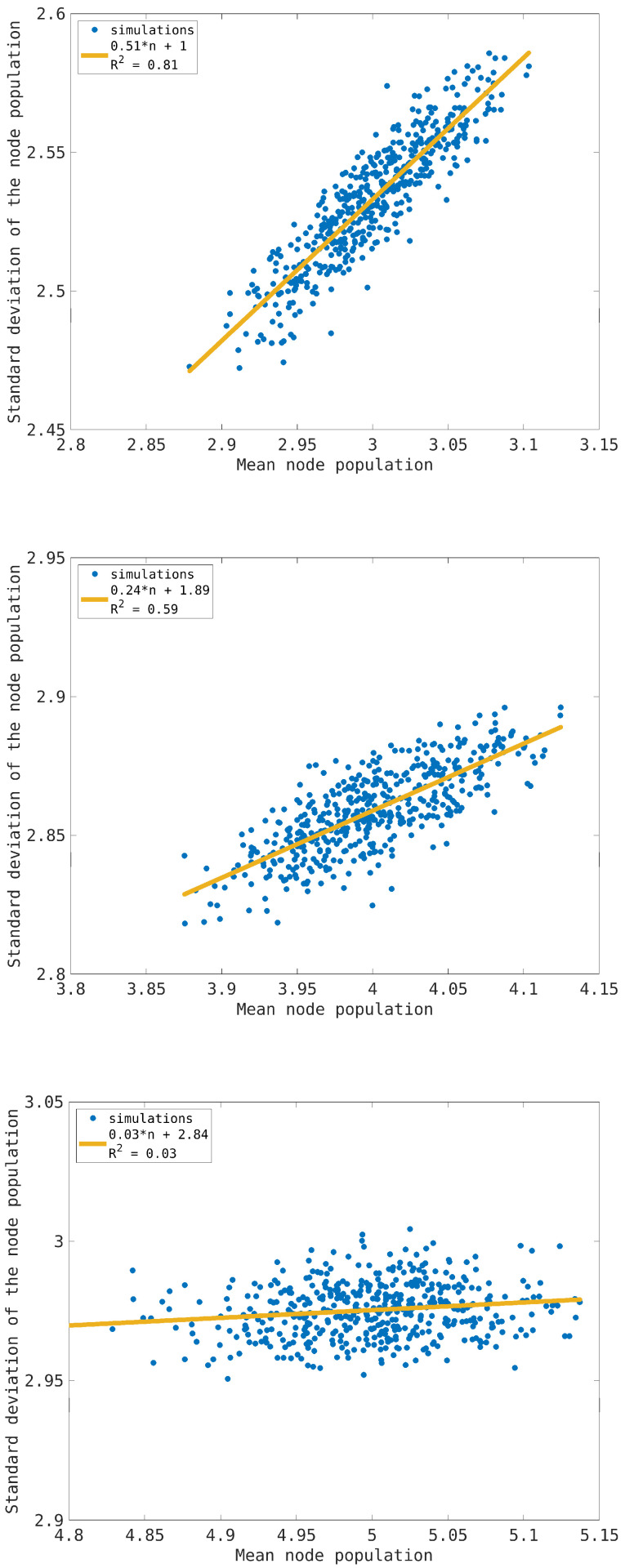
Standard deviation of the single-node traffic load for the transport network model in the synchronous dynamics case as a function of the corresponding mean value. From top to bottom, the figures refer to an average traffic load per node $\bar{n}=3,4,5$ (from top to bottom), and the continuous lines refer to the linear interpolation whose parameters are reported in the insets.

Both these effects are a consequence of the fact that the node degree satisfies d≥2; indeed, the incoming flows can change the node load by up to *d* particles in one time step, so the frequency of the empty state is depressed compared to the one-step process. At the same time, if we have a node with a traffic load $n\in [n^{\max}-1,n^{\max}-d+1]$, at the next time step, its state will be $n\in [n^{\max}+d-1,n^{\max}]$, resulting in overloaded nodes that cannot receive any particles until their state is again $n<n^{\max}$. The frequency of the overloaded node is fast decaying due to the dynamical rules. Even in the synchronous case, the exponential interpolation is a good approximation of the true stationary distribution according to a local maximum entropy principle. To illustrate how the emergence of congestion affects the single-node distribution, we have considered the dependence of the standard deviation on the mean traffic load for single nodes. The results are reported in Figure 3 for the average loads $\bar{n}=3,4,5$. In the cases $\bar{n}=3,4$, we propose a linear interpolation that corresponds to an exponential-like distribution. The point distribution in the pictures reflects the heterogeneity of node behavior in the transport network, which is maximum at the low traffic load $\bar{n}=3$. This correlation is completely lost at the critical load $\bar{n}=5$, when the standard deviation is maximum but the heterogeneity of the nodes is minimum.

**Figure 4:**
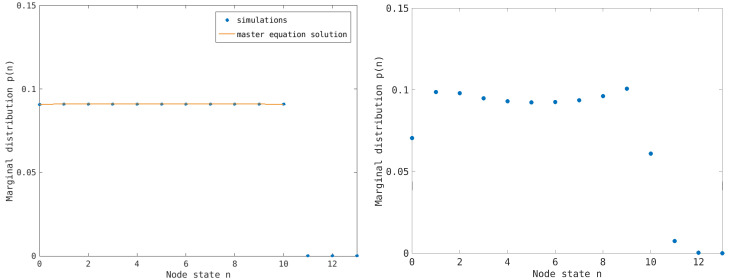
Stationary distribution probability for the transport network model using the same parameters as in Figure 2 at the critical value of the traffic load $\bar{n}=5$. The left picture refers to the one-step process, where the empirical distribution is almost flat (slightly peaked at n=5), whereas the right picture refers to the synchronous dynamics, where the distribution is bimodal near the empty and congested states, but we do not have an analytical approximation.

However, to understand the emergence of congestion, one must also consider the spatial distribution of congested nodes to detect the presence of congested clusters. As previously discussed, synchronous dynamics introduces a correlation between the states of connected nodes when they are empty or congested. This correlation gives rise to entropic forces that tend to cluster the empty and congested nodes. To highlight this effect, we compute the stationary distribution when $\bar{n}=n^{\max}/2$. The results for the considered random network are shown in Figure 4, both for the single-step process and the synchronous dynamics. We observe that, in the first case, the distribution is almost flat, whereas, in the second case, the presence of entropic forces induces a bimodal distribution with peaks at the empty and congested nodes.

**Figure 5:**
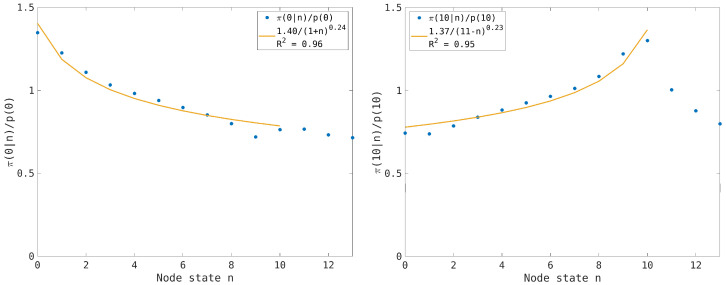
(**Left picture**): The normalized conditional probability π(0|n)/p(0) for the transport network model is numerically computed using the synchronous dynamics (dots). The average traffic load is $\bar{n}=5$. The continuous line is a proposed interpolation with the power law function reported in the inset, together with the corresponding $R^{2}$-value. (**Right picture**): The same as in the left picture for the conditional probability $\pi \left(n^{\max}|n\right)/p\left(n^{\max}\right)$ (dots). The continuous line corresponds to the power law interpolation reported in the inset.

**Figure 6:**
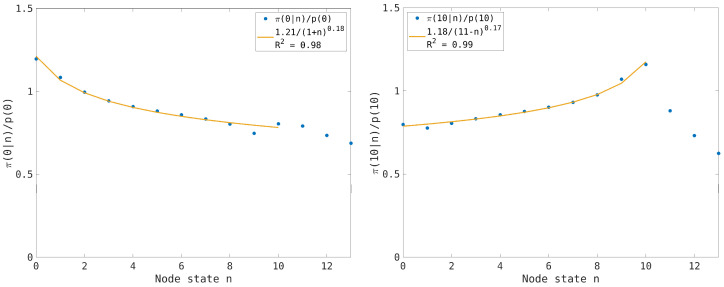
(**Left picture**): The normalized conditional probability π(0|n)/p(0)(dots) is computed (cfr. Figure 5 left) using an average traffic load $\bar{n}=3$. In the inset is reported the power law interpolation (continuous line). (**Right picture**): The normalized conditional probability $\pi \left(n^{\max}|n\right)/p\left(n^{\max}\right)$(dots) is computed (cfr. Figure 5 right) using an average traffic load $\bar{n}=7$. In the inset is reported the power law interpolation (continuous line).

To quantify the entropic forces, we have computed the conditional probabilities π(0|n) and $\pi (n^{\max}|n)$ that denote the probability that a node with load *n* has an empty neighbor or a congested neighbor, respectively. In the case of the single-step process, these probabilities are

$$\pi \left(0|n\right)≃p\left(0\right)\;\pi \left(n^{\max}|n\right)≃p\left(n^{\max}\right)$$

since the nodes evolve independently in the thermodynamic limit. Conversely, in the synchronous case we expect that the correlation among the node states will increase the values π(0|0) and $\pi (n^{\max}|n^{\max})$ near the boundary states n=0 and $n=n^{\max}$. The numerical results are reported in Figure 5 and Figure 6, where the effect of the entropic force is clearly visible at low and high traffic load and an interpolation of the numerical results by a power law distribution is proposed.

(27) $$\begin{array}{cc} \pi (0|n) & \propto \frac{p(0)}{{\left(1+n\right)}^{\alpha }}\\ \pi (n^{\max}|n) & \propto \frac{p(n^{\max}}{{\left(n^{\max}+1-n\right)}^{\alpha }} \end{array}$$

**Figure 7:**
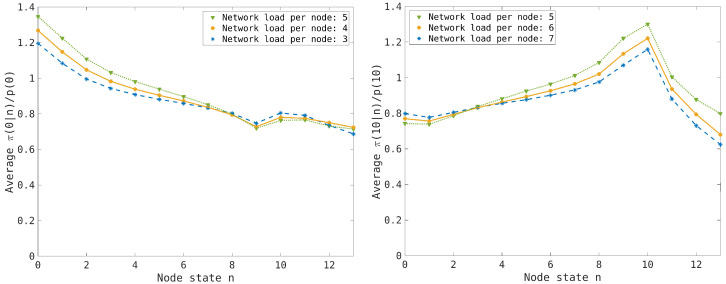
(**Left picture**): Normalized conditional probability π(0|n)/p(0) for the transport network model using the synchronous dynamics for the average traffic loads $\bar{n}=3,4,5$; different symbols are used to distinguish the cases, as reported in the inset. (**Right picture**): The same as in the left picture for the normalized conditional probability π(10|n)/p(10) with average traffic loads $\bar{n}=7,6,5$.

In the numerical simulations, we have initially considered an average traffic load of $\bar{n}=n^{\max}/2$ (Figure 5), where the interpolation (27) of the numerical results highlights the symmetry of the entropic forces when we consider the effect on the empty and the congested nodes. Then, we have simulated a low traffic load, ${\bar{n}}_{l}\ll n^{\max}$, and a high traffic load, ${\bar{n}}_{h}≃n^{\max}$, but with the symmetric condition ${\bar{n}}_{h}=n^{\max}-{\bar{n}}_{l}$ (Figure 6). The interpolation (27) provides almost the same exponent in the two cases consistently with the equivalence of the gap dynamics and the particle dynamics in the case of a balanced network. The case $\bar{n}=n^{\max}/2$ is critical since the exponent is maximum (so one has a faster decaying of the correlation), suggesting that the effect of the two entropic forces tends to balance when the traffic load of the nodes is far from the limit values. This effect is illustrated by Figure 7, where we compute the normalized conditional probabilities π(0|n)|p(0) and $\pi \left(n^{\max}|n\right)/p\left(n^{\max}\right)$ for the critical traffic load $\bar{n}=5$. The results also point out the symmetrical behavior of the particle and gap dynamics (except near the boundary values n=0 and $n\geq n^{\max}$).

To see how the entropic forces depend on the traffic load, we have computed the normalized conditional probabilities π(0|n)/p(0) and $\pi \left(n^{\max}|n\right)/p\left(n^{\max}\right)$ for a traffic load $\bar{n}=3$ (see Figure 6 left) and $\bar{n}=7$ (see Figure 6 right), respectively. The results suggest that the maximum effect of the entropic forces is achieved at the critical load $\bar{n}=n^{\max}/2$, whereas the connected node states tend to be weakly correlated at low or high traffic loads. This can be understood since the probability p(0) increases for low traffic load, decreasing the effect of the entropic force, and similarly it happens to $p(n^{\max}$ at high traffic load. This explains the quite good exponential interpolation of the distribution p(n) for the synchronous case (see Figure 2) that is computed by maximizing the entropy (i.e., considering the nodes independent in the thermodynamic limit).

To illustrate this effect, we compare the normalized conditional probabilities π(0|n)/p(0) and $\pi \left(n^{\max}|n\right)/p\left(n^{\max}\right)$ for traffic loads $\bar{n}=3,4,5$ and $\bar{n}=7,6,5$, respectively. The results are reported in Figure 7, where we observe the entropic forces have a maximum effect at $\bar{n}=5$ and the symmetry of the particles and gap dynamics for the homogeneous transport network.

**Figure 8:**
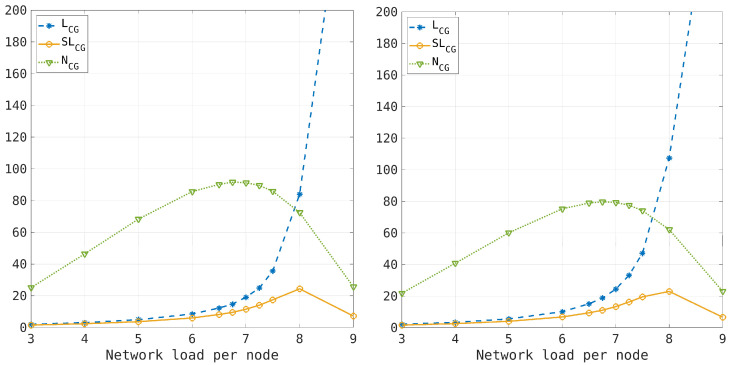
Size of the largest congested cluster ($L_{CG}$ curve with blue dots), size of the second-largest congested cluster ($SL_{CG}$ curve with yellow dots), and number of congested clusters ($N_{CG}$ curve with green dots) as functions of the average traffic load. The **left picture** refers to the one-step process approximation, and the **right picture** refers to the synchronous dynamics. The percolation transition occurs when a macrocluster of congested nodes emerges in the network ($L_{CG}$ curve). The numerical results indicate $\bar{n}≃8$ as the critical traffic load for both cases (the one-step and the synchronous dynamics). At this value, the second-largest cluster curve has a maximum, whereas the maximum value of the number of congested clusters is achieved at $\bar{n}≃7$, suggesting that the merging of small congested clusters precedes the percolation transition.

According to the previous results, the traffic load $\bar{n}=n^{\max}/2$ is a critical value for system fluctuations. However, the emergence of macroscopic congestion depends on the distribution of congested nodes in the transport network as the traffic load increases. The formation of a giant cluster is the fingerprint of the percolation transition, which has been proposed to describe congestion on an urban road network [11]. We have computed the number of congested clusters, the largest cluster size, and the second largest cluster size for the transport network model, considering different traffic loads, both in the case of the one-step process approximation and in the synchronous dynamics. The simulation results are shown in Figure 8, where the percolation transition occurs when the largest cluster size steepens, while both the number of clusters and the second largest cluster size decrease due to the formation of a large congested cluster. The results indicate a percolation threshold when the average traffic load is $7<\bar{n}<8$ for both the one-step process and the synchronous dynamics. The main difference is observed in the number of congested clusters, which is lower in the case of synchronous dynamics. This is consistent with the presence of entropic forces that tend to cluster the congested nodes (thus reducing their number) and result in a larger dimension of the largest cluster in the synchronous case. The coincidence of the percolation threshold in both cases can be explained by the weak effect of entropic forces at high traffic loads. The formation of a giant congested cluster can destroy the connectivity of the transport network (percolation transition), even if the cluster is not static in the model, as the outgoing flow from the congested nodes moves the cluster within the network.

## 6. Conclusions

We adopted a reductionist approach to highlight the universal properties of the congestion transition in an urban road network. Our approach uses a Markov random process on a graph to simulate two fundamental features of a transport network: the existence of a maximum flow rate and the existence of a maximum capacity. Two different dynamics are considered: the one-step process, where a randomly chosen node moves each time step, and the synchronous dynamics, where all nodes move together using the same information on the network state. Our main goal is to highlight universal characteristics of the congestion transition and, in particular, to study the effects of traffic load fluctuations.

Throughout our analysis, we maintained the assumption that the transport network is in a balanced condition (cf. def. (22)), where the average incoming flow at each node equals the average outgoing flow. This condition means that the average flows are in equilibrium, and this is related to the concept of Wardrop equilibrium, where paths are optimized across the network to avoid congestion. In the case of non-balanced transport networks, there exist nodes that inevitably become congested as the traffic load increases and play the role of hot spots for congestion formation. Conversely, if the balance condition is satisfied, congestion is driven by traffic load fluctuations. Our analytical methods, particularly the application of a maximum entropy principle, allowed us to characterize the distribution of fluctuations across nodes and identify how congestion emerges in a continuous way as the overall traffic load increases. Notably, we study the entropic forces in synchronous dynamics, leading to the clustering effect of congested and empty nodes.

The stationary distribution of traffic load fluctuations can be approximated by a local entropy principle, and the congestion transition is characterized by a peak in the traffic load variance of the nodes, yet without singularity in the thermodynamic limit. Numerical simulations further demonstrated the formation of a macroscopic congested cluster, resembling a percolation-like transition within the network, requiring traffic loads nearing maximum capacity for its emergence. The simulations highlight the effect of the entropic forces in the synchronous process on the congestion transition as a greater dimension of the congested clusters compared to the one-step process, but these slightly affect the critical traffic load for the percolation transition.

The considered model is certainly a simplification of the microscopic urban traffic complexity, but it allows us to underscore some universal macroscopic features. Our results explain the emergence of congestion as a continuous process that creates clusters of congested nodes mimicking the percolation transition (i.e., the formation of a macroscopic congested cluster at high traffic load). The existence of entropic forces depends on the network dynamics and favors the cluster formation of empty and congested nodes. This research not only provides insights into the nature of traffic congestion but also contributes to the theoretical framework necessary for enhancing urban transportation systems.

The application of control strategies to mitigate the congestion effect requires two further steps. On one hand, the time scales of congestion formation related to the relaxation time scales of the system need to be studied to estimate congestion diffusion in the transport network. On the other hand, strategies must be developed to reduce the fluctuation variance of the local traffic load across the whole network using intersection dynamics. A quantitative application of our results to real transport networks certainly requires more complex models, but we do not expect new physical phenomena. The possibility of performing a validation process needs datasets containing information on traffic load fluctuations at a road scale. This could be possible using GPS datasets with a representative sample of vehicle trajectories on homogeneous road networks, but at the moment they are not publicly available.

## Data Availability

The original contributions presented in the study are included in the article, further inquiries can be directed to the corresponding author.

## Abbreviations

The following abbreviations are used in this manuscript:

OD	Origin Destination
MEP	Maximum Entropy Principle
DB	Detailed Balance

## Appendix A. Analysis of the Stationary Distributions

Using a dynamical point of view, the stationary distribution is proportional to the visiting frequency of any microstate during the evolution when the ergodic property holds. The normalizing constant in Equation (11), $C_{N}^{n^{\max}}\left(\vec{p}\right)$, can be defined in a recursive way using a generating function,

(A1) $$C_{N}^{n^{\max}}\left(\vec{p}\right)=\sum_{|n|=N}^{n^{\max}}\prod_{i=1}^{M}p_{i}^{n_{i}}≃\frac{1}{N!}{\left.\frac{d^{N}}{dx^{N}}\right|}_{x=0}\prod_{i=1}^{M}\frac{1}{\left(1-p_{i}x\right)},$$

when $n^{\max}\gg 1$. An analytical estimate of the normalizing constant is obtained using the Cauchy theorem

$$C_{N}\left(\vec{p}\right)\leq \prod_{i=1}^{M}\frac{1}{1-p_{i}}.$$

The function

$$g\left(x\right)=\prod_{i=1}^{M}\frac{1}{1-p_{i}x}$$

is analytic in the disk |x|≤1, and it has poles at $x=p_{i}^{-1}$, so that the radius of convergence, $R_{*}$, of the Taylor expansion at x=0 depends on the nearest pole to the origin: $R_{*}=\min_{i=1,...,M}\left\{p_{i}^{-1}\right\}$. We can see that if the distribution $p_{i}$ is not uniform, $C_{N}\left(\vec{p}\right)$ is dominated by the contribution of the greatest $p_{i}$.

Let $p_{i}=M^{-1}$, then all the microstates have the same weight and we have a simple solution for the partition function, $C_{N}\left(p\right)$, that corresponds to the number $C_{N}\left(M\right)$ of *M*-component vectors, $\vec{n}$, with non-negative integer vectors such that $|\vec{n}|=N$,

(A2) CN(M)=1MNN+M−1M−1.

The stationary distribution becomes

(A3) ρs(n→)=N+M−1M−1−1,

and it maximizes the Gibbs Entropy (15) by definition. Since this is an attractive distribution, we expect that the one-step process (7) increases the entropy during the evolution. We remark that for *N* finite, the state of the nodes are not independent since the state of each node affects the other node states, but they can be considered independent for N→∞. The empty state is a boundary condition state at which the outgoing flow from the node is zero. If one randomly chooses the network state $\vec{n}$, the probability that a node has *n* particles requires estimation of the mean value,

$$\frac{♯\{|\vec{n}|=N,n_{k}=n,k=1,...,M\}}{M}=\frac{1}{M}\sum_{k}\delta_{n,n_{k}}.$$

One can compute the average value,

$$E\left(\delta_{n,n_{k}}\right)=\sum_{|\vec{n}|=N,n_{k}=n}\rho_{s}\left(\vec{n}\right),$$

and if the balance condition holds we get

$$\sum_{|\vec{n}|=N,n_{k}=n}\rho_{s}\left(\vec{n}\right)=\frac{C_{N-n}\left(M-1\right)}{C_{N}\left(M\right)}.$$

It follows that

E(δn,nk)=N−n+M−2M−2N+M−1M−1−1=(M−1)N...(N−n+1)(N+M−1)...(N−n+M−1),

and for N,M≫1, we estimate

$$\frac{♯\{|\vec{n}|=N,n_{k}=n\}}{M}≃\frac{1}{1+\bar{n}}\frac{1}{{\left(1+{\bar{n}}^{-1}\right)}^{n}}.$$

Then, a direct calculation shows

$$\frac{1}{1+\bar{n}}\sum_{n\geq 0}\frac{1}{{\left(1+{\bar{n}}^{-1}\right)}^{n}}=\frac{1}{1+\bar{n}}\frac{1+{\bar{n}}^{-1}}{{\bar{n}}^{-1}}=1,$$

and the average value is

$$\begin{array}{cc} E(n) & =\frac{1+{\bar{n}}^{-1}}{1+\bar{n}}\sum_{n\geq 1}\frac{n}{{\left(1+{\bar{n}}^{-1}\right)}^{n+1}}=-\frac{1}{\bar{n}}\frac{d}{d{\bar{n}}^{-1}}\sum_{n\geq 0}\frac{1}{{\left(1+{\bar{n}}^{-1}\right)}^{n}}\\ \; & =-\frac{1}{\bar{n}}\frac{d}{d{\bar{n}}^{-1}}\left(1+\bar{n}\right)=\frac{1}{{\bar{n}}^{-1}}=\bar{n} \end{array}$$

since the nodes are all equivalent. In the thermodynamic limit N→∞, the marginal distribution for a single node, $p_{k}\left(n\right)$, reads

(A4) $$p_{k}\left(n\right)=\frac{{\left(1+\bar{n}\right)}^{-1}}{{\left(1+{\bar{n}}^{-1}\right)}^{n}}≃\frac{1}{\bar{n}}e^{-n/\bar{n}},$$

where the last estimate requires that $\bar{n}$ is not too small. The marginal distribution $p_{k}\left(n\right)=p\left(n\right)$ gives the probability to observe the state *n* for any node, and it describes the distribution of the node states in a large network N≫1. This distribution is also associated with the fluctuations of the node state with respect to the mean value $\bar{n}$ (but the time scale of the fluctuations depend on the spectral properties of the Laplacian matrix of the master Equation (7). Since the stationary distribution of the network state maximizes the entropy, the same is expected for the marginal distribution with the constraints on the state of the *k*-node and the total number of particles. Indeed, in the thermodynamic limit we have the maximal entropy solution with $E\left(n\right)=\bar{n}$ for an ensemble of equiprobable microstates, and the state of the *k*-node becomes independent from the states of all the other nodes. We remark that this result holds for any random walk on graphs in a balance condition. In such a case, the dynamics are dominated by the fluctuations that scale as $\bar{n}$ (not $\sqrt{\bar{n}}$ as for the Poisson distribution). The nodes with a population below the average value are over-expressed, and few nodes have a great population, but the fluctuations continuously change the state of each node redistributing the particles.

In the one-step process evolution, the Markov process associated with the master Equation (7) is realized by randomly choosing a link j→i in the graph and, if $n_{j}>0$, moves a particle with probability $\pi_{ij}\Delta t$. Then, the network state changes and another movement is performed. The counterpart of the one-step process is synchronous dynamics, when at each time step all the nodes can move a particle if their state is not empty. For a given state, $\vec{n}$, the outgoing flows are

$$\Phi^{-}\left(\vec{n}\right)=\sum_{j_{1},...,j_{M}=1}^{M}\pi_{j_{1}1}\left(n_{1}\right)...\pi_{j_{M}M}\left(n_{M}\right)\rho \left(\vec{n},t\right),$$

where one considers all the possible exchanges among the nodes so that many of the terms are zero in the previous sum. However, according to the definition of the transition rates, $\pi_{ij}\left(n\right)$, it is necessary to extend the definition by setting $\pi_{ii}\left(0\right)=1$, otherwise the product that defines the probability rate vanishes each time a configuration contains a null node. In a similar way, the incoming flow can be written in the form

(A5) $$\begin{array}{ccc} & & \Phi^{+}\left(\vec{n}\right)=\sum_{j_{1},...,j_{M}=1}^{M}E_{j_{1}}^{-}...E_{j_{M}}^{-}E_{1}^{+}...E_{M}^{+}\pi_{j_{1}1}\left(n_{1}\right)...\pi_{Mj_{M}}\left(n_{M}\right)\rho \left(\vec{n},t\right)\\ & & =\sum_{j_{1},...,j_{M}=1}^{M}\pi_{j_{1}1}\left(n_{1}-m_{1}+1\right)...\pi_{j_{M}M}\left(n_{M}-m_{M}+1\right)\rho \left(\vec{n}+\vec{1}-\sum_{k}{\hat{e}}_{j_{k}},t\right), \end{array}$$

where $m_{k}$ counts the repetitions number of the index *k* in the *M*-nuple $j_{1},..,j_{M}$: if $n_{k}-m_{k}+1$ is the initial configuration of the *k* node, it has to receive $m_{k}$ particles to obtain the final configuration, $n_{k}$. We remark that, in synchronous dynamics, all the nodes move referring to the same network state, since the state updates after all the displacements. An analytic solution of Equation (A5) is not available.

In a balance condition, one considers all the possible exchanges among the connected nodes, assuming that all the nodes are not empty, and using the transition probability rates one has $<m_{k}>=1$ (the average incoming flow equals the outgoing flow). If a node is connected to empty nodes, the incoming flow reduces (on average), whereas the outgoing flow is fixed. This means that there exists an effective ’force’ that attracts the node state to the zero state when it is connected to empty nodes. Therefore, a node gets to a neutral equilibrium state when it is connected to non empty nodes and the balance condition holds. However, each time one of its neighborhoods is empty, there is a negative average net flux. The converse is also true, if we consider a node in a zero state: the net average flux is always positive and it is maximum when all the connected nodes are not empty. This effect gives an explanation to the exponential limit distribution as the solution of a diffusion process with a boundary conditions and there exists a constant force toward the boundary.

We analyze the node dynamics near the zero state in detail, but a similar argument can apply to the nodes near the congested state. If $n^{\max}\gg 1$, the stationary condition for the flows at a given node is

$$\left(1-p_{0}\right)\sum_{j}\pi_{ij}=\sum_{j}\pi_{ji}\left(1-p_{0}\right),$$

which is always satisfied in a balance condition when the probability of the zero state, $p_{0}$, is the same for all the nodes. The average number of zero states in the stationary distribution is a property of the dynamics. In the one-step process dynamics, all the configurations are equiprobable, and to compute the probability to observe a certain number of zero states one has to count the number of possible configurations distributing the zero states in the network. In the thermodynamic limit, the fraction of zero states can be computed using the distribution (A4) when the nodes can be considered independent. In synchronous dynamics, the presence of the zero states reduces the expected incoming flows to the boundary nodes, i.e., the nodes connected to the empty nodes. A node $k_{b}$ is a boundary node of a given network state $\vec{n}$ ($k_{b}\in B\left(\vec{n}\right)$) if there exists *j* s.t. $\pi_{k_{b}j}\neq 0$ with $n_{j}=0$. Let $M_{0}$ be the number of empty nodes of the state $\vec{n}$,

$$M_{0}=♯\{j:|\vec{n}\left|=N,\;n_{j}=0\;j=1,...,M\right\}.$$

The number of boundary nodes depends on the distribution of the zero states in the network. The number of the boundary nodes is the volume, $V=♯B(\vec{n})$. It is clear that the volume, $V_{0}\left(\vec{n}\right)$, is minimum when the zero state nodes form a cluster. In the evolution of the transport network model, the nodes at the boundary feel an effective force that decreases their state toward zero. If $V_{0}\left(\vec{n}\right)$ is minimum, the total ‘force’ acting on the node states at a given time is also minimum, and this corresponds to an ‘equilibrium situation’. Therefore, we expect that these configurations are favored in the stationary condition. According to this point of view, we call these forces ‘entropic’ since they change the microstate probability favoring the state with minimum boundary volume, $V_{0}\left(\vec{n}\right)$. This effect is counteracted by fluctuations due to the stochastic dynamics that increase the configuration entropy distributing the particles among the nodes, so that the effect of entropic force decreases when the node degree increases. Since the entropic forces in the synchronous dynamics decrease the total entropy of the stationary distribution, the non-reversibility character implies that one has a non-equilibrium stationary state with an entropy production. An open question is if the stationary state can be characterized by a minimum entropy production [27].

The effect of the entropic forces on a node depends on its connectivity: a node with a high degree is little affected by having an empty neighbor, and we expect a clustering effect of zero state nodes that have a low incoming degree. In a highly connected graph, the entropic forces are negligible and one can approximate the invariant measure by a uniform measure as in the one-step process.

For a given number of zero state nodes, the configuration with a minimum boundary volume depends on the graph structure; in principle, this could be related to the existence of a community that is poorly connected with the rest of the network. This problem requires one to define a geometry for the network to understand how empty node clusters appear and increase in the transport network model.
